# Opening the gateway to enhanced *Eucalyptus*

**DOI:** 10.1186/1753-6561-5-S7-I19

**Published:** 2011-09-13

**Authors:** Ziv Shani

**Affiliations:** 1 Futuragene Ltd., Rechovot, Israel

## 

Eucalyptus is the second most planted tree on earth. Despite this ranking, its mature breeding programs, clonal propagation and deployment and short rotation time make it a leading target for biotechnology improvement. The recently completed eucalyptus genome project provides an enormous opportunity for understanding and developing new traits for eucalyptus forestry. The main obstacle for widespread trait enhancement remains the recalcitrance of commercial eucalyptus clones to transformation and thus the lack of a suitable prototyping system for this species. We believe that the future success of trait development in eucalyptus will be enhanced by close collaboration between academic centers and industrial partners. Some of FuturaGene’s models for academic collaboration will be described in this presentation.

An example of such potential collaborations may involve BRASUZ1, which constituted the germplasm base for the eucalyptus genome project. This key clone was donated by Suzano Pulp and Paper, FuturaGene’s parent, to the genome project. We will present our progress with the transformation protocol for BRASUZ1, which could provide a platform for collaborations. We will also present our perspective on the development of improved transgenic eucalyptus, and an outline for such development programs.

